# Persistently high estimates of late night, indoor exposure to malaria vectors despite high coverage of insecticide treated nets

**DOI:** 10.1186/1756-3305-7-380

**Published:** 2014-08-20

**Authors:** M Nabie Bayoh, Edward D Walker, Jackline Kosgei, Maurice Ombok, George B Olang, Andrew K Githeko, Gerry F Killeen, Peter Otieno, Meghna Desai, Neil F Lobo, John M Vulule, Mary J Hamel, Simon Kariuki, John E Gimnig

**Affiliations:** Centre for Global Health Research, Kenya Medical Research Institute, Kisumu, Kenya; Department of Microbiology and Molecular Genetics, Michigan State University, East Lansing, MI USA; Environmental Health & Ecological Sciences, Ifakara Health Institute, Dar es Salaam, United Republic of Tanzania; Vector Biology Department, Liverpool School of Tropical Medicine, Liverpool, UK; Division of Parasitic Diseases and Malaria, Centers for Disease Control and Prevention, Atlanta, GA USA; Department of Biological Sciences, University of Notre Dame, Notre Dame, IN USA

**Keywords:** *An. gambiae*, *An. funestus*, Insecticide treated nets, Behavior

## Abstract

**Background:**

It has been speculated that widespread and sustained use of insecticide treated bed nets (ITNs) for over 10 years in Asembo, western Kenya, may have selected for changes in the location (indoor versus outdoor) and time (from late night to earlier in the evening) of biting of the predominant species of human malaria vectors (*Anopheles funestus*, *Anopheles gambiae* sensu stricto, and *Anopheles arabiensis*).

**Methods:**

Mosquitoes were collected by human landing catches over a six week period in June and July, 2011, indoors and outdoors from 17 h to 07 h, in 75 villages in Asembo, western Kenya. Collections were separated by hour of the night, and mosquitoes were identified to species and tested for sporozoite infection with *Plasmodium falciparum.* A subset was dissected to determine parity. Human behavior (time going to bed and rising, time spent indoors and outdoors) was quantified by cross-sectional survey. Data from past studies of a similar design and in nearby settings, but conducted before the ITN scale up commenced in the early 2000s, were compared with those from the present study.

**Results:**

Of 1,960 *Anopheles* mosquitoes collected in 2011, 1,267 (64.6%) were morphologically identified as *An. funestus*, 663 (33.8%) as *An. gambiae* sensu lato (*An. gambiae s.s.* and *An. arabiensis* combined), and 30 (1.5%) as other anophelines. Of the 663 *An. gambiae* s.l. collected, 385 were successfully tested by PCR among which 235 (61.0%) were identified as *An. gambiae* s.s. while 150 (39.0%) were identified as *An. arabiensis.* Compared with data collected before the scale-up of ITNs, daily entomological inoculation rates (EIRs) were consistently lower for *An. gambiae* s.l. (indoor EIR = 0.432 in 1985–1988, 0.458 in 1989–1990, 0.023 in 2011), and *An. arabiensis* specifically (indoor EIR = 0.532 in 1989–1990, 0.039 in 2009, 0.006 in 2011) but not *An. funestus* (indoor EIR = 0.029 in 1985–1988, 0.147 in 1989–1990, 0.010 in 2009 and 0.103 in 2011). Sporozoite rates were lowest in 2009 but rose again in 2011. Compared with data collected before the scale-up of ITNs, *An. arabiensis* and *An. funestus* were more likely to bite outdoors and/or early in the evening (p < 0.001 for all comparisons). However, when estimates of human exposure that would occur indoors (π_i_) or while asleep (π_s_) in the absence of an ITN were generated based on human behavioral patterns, the changes were modest with >90% of exposure of non-ITN users to mosquito bites occurring while people were indoors in all years. The proportion of bites occurring among non-ITN users while they were asleep was ≥90% for all species except for *An. arabiensis.* For this species, 97% of bites occurred while people were asleep in 1989–1990 while in 2009 and 2011, 80% and 84% of bites occurred while people were asleep for those not using ITNs. Assuming ITNs prevent a theoretical maximum of 93.7% of bites, it was estimated that 64-77% of bites would have occurred among persons using nets while they were asleep in 1989–1990, while 20-52% of bites would have occurred among persons using nets while they were asleep in 2009 and 2011.

**Conclusions:**

This study found no evidence to support the contention that populations of *Anopheles* vectors of malaria in Asembo, western Kenya, are exhibiting departures from the well-known pattern of late night, indoor biting characteristic of these typically highly anthropophilic species. While outdoor, early evening transmission likely does occur in western Kenya, the majority of transmission still occurs indoors, late at night. Therefore, malaria control interventions such as ITNs that aim to reduce indoor biting by mosquitoes should continue to be prioritized.

## Background

Insecticide treated nets (ITNs) are one of the primary tools for malaria prevention and control and are being widely scaled up in sub-Saharan Africa (SSA). Between 2000 and 2012, household ownership of ITNs in SSA rose from 3% to 56%. The scale up of vector control along with improved case management practices has resulted in decreases in malaria incidence and mortality. Since 2000, the incidence of malaria declined by 29% and mortality due to malaria declined by 45% [1].

However, these gains are threatened by the development of physiological or behavioral resistance in the malaria vectors. Physiological resistance to pyrethroid insecticides used on ITNs has been widely documented [2] although evidence that such resistance is compromising ITN effectiveness is limited [3]. Behavioral resistance may be a greater threat to the effectiveness of ITNs [4, 5]. ITNs are effective for preventing malaria because many malaria vectors feed late at night while people are asleep [6–8]. Should ITNs select for mosquito species or sub-populations, which tend to feed outdoors or early in the evening, their effectiveness may be limited. During large scale trials of ITNs, evidence for shifts in biting behavior was mixed with reports of earlier biting in some trials [9, 10] but not others [11, 12]. As ITNs and indoor residual spraying (IRS) have been scaled up in sub-Saharan Africa, there are reports of shifting mosquito behaviors to proportionately more outdoor biting in Tanzania [13] and Bioko Island [14]. In west Africa, *An. funestus* has been observed biting into the morning as late as 11 h, well after most people would have awoken and emerged from under their nets [15, 16]. In contrast, high exposure to indoor biting mosquitoes late at night was observed in six sites in sub-Saharan Africa, even among users of ITNs [17]. However, even in areas where shifts in mosquito behavior do not occur, residual biting that occurs outdoors or early in the evening before humans have retired under ITNs may be adequate to maintain malaria transmission [18, 19].

The Asembo Bay area of western Kenya was the site of a large scale, cluster randomized ITN trial in the late 1990s [20, 21]. After the trial, ITNs were provided to all villages and household ownership of any net was maintained at over 90% through a routine retreatment program and periodic net replacement campaigns until 2007. Since the early 2000s, national coverage of ITNs in Kenya was increased through multiple channels, including routine distribution through antenatal and child welfare clinics, social marketing and mass campaigns in 2006 and 2011. Household ownership rose from <5% to 48% in 2010 and was 60% in the lake endemic region [22]. As ITNs were scaled up and national malaria treatment policy shifted from chloroquine and SP to artemisinin based combination therapies [23], the burden of malaria in western Kenya declined substantially. The prevalence of *Plasmodium falciparum* infection among children <5 years of age in Asembo fell from >80% in the 1990s [24] to <30% in 2008. However, prevalence rose to over 40% in 2009 and remained stagnated in subsequent years [25]. Similar trends were observed in neighboring areas within western Kenya [26].

One potential explanation for the persistently high malaria in this area is changing behaviors of malaria vectors, which result in mosquitoes avoiding the insecticidal effects of ITNs. This study was designed to test the hypothesis that the increase in malaria in western Kenya was due to changing behaviors of the primary malaria vectors to bite in places or times when people were less likely to be under their insecticide treated nets.

## Methods

### Study site and population

This study was conducted in western Kenya through an on-going collaboration between the Kenya Medical Research Institute and the Centers for Disease Control and Prevention. The study was conducted in Asembo, Rarieda District in western Kenya covering approximately 200 km^2^ of gently rolling hills bisected by small streams that discharge into Lake Victoria. Asembo is a rural region characterized by high, year-round transmission of malaria. There are two main periods of high rainfall: March to June (the long rains) and October-November (the short rains) which are typically associated with peaks of malaria transmission. The population of Asembo is mainly comprised of subsistence farmers residing in clusters of houses (compounds) scattered across the landscape, interspersed with farmland and slow streams. The houses vary from traditional mud huts with thatched roofs to brick houses with iron sheet or tiled roofs. Most of the houses have open eaves, which are important entry and exit points for mosquitoes. The total population of Asembo was estimated at 65,190 in 2010.

The annual entomological inoculation rate (EIR) in the area in the early 1990s was estimated at over 300 infectious bites per person per year [27]. Before the late 1990s, net use was rare throughout western Kenya. However, in the late 1990s, after distribution of ITNs to residents of Asembo during a large scale randomized trial, populations of vector species diminished and entomological measures of transmission declined by ~90% [28]. Estimates of annual EIRs using light traps or pyrethrum spray catches since 2003 remained below 15 infectious bites per person per year (M. N. Bayoh, unpublished data). Since the randomized trial, ITNs have been scaled up throughout western Kenya through various strategies including routine distribution to high risk groups, social marketing and mass distribution campaigns in 2006 and 2011. By 2008, 64% of children below five years of age were reported to have slept under an ITN the previous night [25]. In 2011, just after the mass distribution campaign, use of any net was 88.8% and use of an ITN was 74.3% (M. Desai, unpublished data). Over the past 2 decades, encompassing the time during which ITNs appeared in the Asembo community, populations of the main malaria vector species (*Anopheles gambiae* s.s., *Anopheles funestus* and *Anopheles arabiensis*) declined dramatically and there were changes in the relative proportions of each species collected inside houses. In the mid to late 2000s, *An. arabiensis* became the predominant vector with very low proportions of both *An. gambiae* and *An. funestus*[29]. Recently, the population of *An. funestus* in Asembo has resurged [30].

### Recruitment of mosquito collectors

The human landing catches (HLCs) were conducted for 24 nights over 6 weeks in June-July 2011, which corresponded to the peak transmission season in western Kenya. In each of the 75 villages of Asembo included in the study, two males aged 18 or older were hired as collectors. Selection criteria included willingness and consent to work as collectors, permanent residency in the village, and experience in entomological projects in the area or known to study staff as reliable. Collectors were provided with a treatment dose of artemether-lumefantrine to clear any parasites as well as daily prophylaxis with atovaquone-proguanil and were tested for malaria every 2 weeks.

### Human landing catches

Collectors were organized into 38 teams, each team comprised of 4 individuals from 2 neighboring villages with the exception of one team that consisted of 2 collectors serving one village. Collections were rotated among the houses of collectors within each team such that all their houses were completed in a week. All the 4 member teams collected from a different house each night for 4 nights while the 2 member team collected from only 2 different houses, thus doing one house per night for 4 nights. Collections began at 17 h and ended at 7 h the next day. The collection period was divided into two 7-hour shifts; an early shift from 17 h until midnight and a late shift from midnight until 7 h hours both inside and outside the house. Each collector was assigned a shift on each day of collection. At each site, the early shift collectors were retired at midnight and replaced by the late shift collectors. To perform the HLC, each collector sat in a chair with their lower legs exposed to their knees and collected mosquitoes that landed on their lower legs using a mouth aspirator. Collected mosquitoes were transferred to a labeled paper cup and provided with cotton soaked in sugar water. New cups were used for each hour and each location and each cup was labeled with the site of collection, the collection hour and the location of collection (indoors or outdoors). Collections were carried out for 45 minutes during each hour with a 15 minute break before resuming collections for the next hour. In the event of rainfall during collection, outdoor collectors were given the discretion to stop collecting and indicate the respective hours when collections were interrupted. Indoor collectors were instructed to continue regardless of rainfall. Outdoor collections were interrupted in 6.5% of person-hours of collections. However, since some mosquito collections occurred during part of these hours, subsequent analyses were not adjusted for any stoppage of the collections.

### Supervision of collectors

Collection teams were contacted each night by a mobile phone call to determine who was working and if there were any problems. Each team was contacted 4–5 times throughout the night at randomly selected hours during the early and late shifts making sure the calls were unpredictable. In addition to the calls, a team of supervisors performed random spot checks on 3–4 teams per night to ensure that the collections were going on as directed.

### Laboratory analysis

All mosquitoes collected were identified to species morphologically [6, 7] and by PCR for the identification of *An. gambiae s.l.* to sibling species level [31]. Individual mosquitoes were tested for *Plasmodium falciparum* sporozoites by sandwich ELISA [32]. A subset of mosquitoes were dissected and ovaries examined to determine their parity status [33].

### Human behavior patterns

In July/August 2011, as part of an annual household survey, a sample of 701 people living in 158 compounds in Asembo were selected for interviews. The total population of Asembo was estimated in 2010 to be 65,190 people living on 11,532 compounds. The 158 compounds were selected out of a total of 5,571 compounds with children <5 years of age by systematic random sampling. As part of the survey, participants were asked about ITN use as well as when they went inside in the evenings, when they went to sleep, when they woke up and when they went outside in the morning.

### Statistical analysis

The entomological inoculation rate for the 6 weeks of HLCs was estimated by summing the numbers collected over each night and each site of collection and calculating an average number of bites per person per night for each location of collection (indoors or outdoors). This was divided by 0.75, as collectors worked for 45 minutes each hour and then multiplied by the sporozoite infection rate estimated using ELISAs. Though a formal statistical analysis was not possible, the daily biting rates and EIRs were compared to other studies conducted using HLC in this area in the past. Data from Beier et al. [27] from 1985 to 1988 were included from Saradidi which is located in the Asembo area. For Miwani and Ahero villages, which were approximately 60 km east of Asembo, EIRs were estimated by converting the total number of mosquitoes biting reported by Githeko et al. [34] to nightly biting rates and multiplying by the sporozoite rates reported by Githeko et al. [35]. Sporozoite rates were not reported by location and were therefore assumed to be the same indoors and outdoors for 1989–90. The predominant species at Ahero were *An. arabiensis* and *An. funestus* while *An. gambiae* s.s. was largely absent. *Anopheles funestus* was also present at Miwani while *An. gambiae* s.l. were reported as 55% *An. gambiae* s.s. and 45% *An. arabiensis*. References used in the comparisons across years for both EIR and biting times are shown in Table 1.

**Table 1:** **Summary of data included in historical comparisons**

Year	Location	Species	Sampling method	Collection frequency	EIR	Hourly biting	Reference
1985-1988	Asembo	*An. gambiae* s.l., *An. funestus*	2 persons per station, one served as bait while the other collected mosquitoes	Weekly for 33 months	Yes	No	[27]
1989-1990	Ahero	*An. arabiensis, An. funestus*	2 persons per station, one served as bait while the other collected mosquitoes	Twice per month for 12 months	Yes*	Yes	[34]
1989-1990	Miwani	*An. gambiae* s.l.	2 persons per station, one served as bait while the other collected mosquitoes	Twice per month for 12 months	Yes*	Yes	[34]
2009	Asembo	*An. arabiensis*	1 person per station, mosquitoes collected from his exposed legs	6 nights per week for 5 weeks during peak transmission	Yes	Yes	[17] & Current
2011	Asembo	*An. gambiae* s.l., *An. gambiae* s.s. *An. arabiensis An. funestus*	1 person per station, mosquitoes collected from his exposed legs	4 nights per week for 6 weeks during peak transmission	Yes	Yes	Current

*EIRs were calculated using sporozoite rates reported by Githeko et al. [35].

Biting times were compared with HLC data from 1989–1990 in Miwani and Ahero [34] and from 2009 in Asembo [17]. In 1989/1990, collections were carried out in each village, by two men twice per night for 12 months. In 2009, collections were rotated among 3 houses in a single village for 30 days in June, the period of peak transmission. In Miwani in 1989/1990, the proportion of *An. gambiae* s.l. that were identified as *An. gambiae* s.s. and *An. arabiensis* was reported as 55% and 45% respectively while Ahero was reported to be exclusively *An. arabiensis*. In 2009, >90% of *An. gambiae* s.l. collected in Asembo were identified as *An. arabiensis*. Biting times were categorized as 18 h to 21 h, 21 h to 24 h, 0 h to 3 h and 3 h to 6 h. Differences in biting times between years were compared by χ^2^ tests. Separate tests were done for each species. Because previous studies did not include collections from 17 h to 18 h or from 6 h to 7 h, data collected during these times in 2011 were not included in the statistical comparisons. Similarly, differences in the proportion of indoor versus outdoor biting were compared between years by χ^2^ tests. Sporozoite rates and parity rates by time and year were also compared using χ^2^ tests. For these comparisons, all *An. gambiae* s.l. mosquitoes collected in 1998/1990 were assumed to be *An. gambiae* s.s. and compared to this species in 2011. For 2009, all *An. gambiae* s.l. were assumed to be *An. arabiensis*. Estimates of exposure to biting mosquitoes in relation to human behavior were estimated using the methods of Seyoum et al. [36]. Human behavioral patterns were estimated from the 2011 survey and assumed to be the same for all years and locations. From these calculations, estimates were derived of the proportion of bites occurring while people were indoors (π_i_) and the proportion of bites occurring while people were asleep (π_s_). Separate estimates of π_i_ and π_s_ were obtained for ITN users and non-users. Exposure among users of ITNs was assumed to be reduced by 93.7% as estimated for East African *An. gambiae* and *An. funestus* by Okumu et al. [37]. For 2011, all hours of collection were used in the estimates of π_i_ and π_s_, including data for the hours 17 h-18 h and 6 h-7 h.

### Ethical approval

This study was approved by the National Ethical Review Committee of the Kenya Medical Research Institute and by the Institutional Review Boards of the US Centers for Disease Control and Prevention and Michigan State University. Written informed consent was obtained from all collectors.

## Results

### Species distribution

A total of 1,960 *Anopheles* mosquitoes were collected in 899 person-nights indoors and 900 person-nights outdoors; of these 1,267 (64.6%) were morphologically identified as members of the *An. funestus* Group, 663 (33.8%) as *An. gambiae* s.l, and 30 (1.5%) as other anophelines (*An. coustani*, *An. ziemanni*, *An. rufipes*). Of the 663 *An. gambiae* s.l. collected, 597 were identified to species by PCR. Of these, 385 (64.5%) were successfully tested by PCR among which 235 (61.0%) were identified as *An. gambiae,* while 150 (39.0%) were identified as *An. arabiensis*. The remaining 212 that did not amplify were combined with the 66 that were not tested by PCR and were considered separately as *An. gambiae* s.l. in subsequent analyses. The *An. funestus* Group mosquitoes were not further identified to species. However, subsequent PCR of mosquitoes morphologically identified as members of the *An. funestus* Group in the Asembo area have all been found to be *An. funestus* s.s. We therefore refer to these mosquitoes as *An. funestus* hereafter.

### Biting rates, sporozoite rates and EIRs

The indoor and outdoor biting rates, sporozoite rates and EIRs are presented in Tables 2 and 3, respectively. Estimated biting rates from 1985–1988 and 1989/1990 are presented for comparison. In 2011, indoor biting rates were approximately 0.2 bites per person per night for *An. gambiae* s.l. or *An. gambiae* s.s., 0.141 bites per person per night for *An. arabiensis* and 1.262 bites per person per night for *An. funestus* (Table 2). Except for *An. funestus,* biting rates were lower in 2009 and 2011 compared to previous years. Indoor biting rates of *An. gambiae* s.l., *An. gambiae* s.s. and *An. arabiensis* were >80% lower in 2009 and 2011 compared to biting rates estimated before 2000. For *An. funestus*, biting rates were actually higher in 2009 and 2011 compared to those observed in 1985 to 1988. Compared to Miwani in 1989/1990, biting rates in Asembo were 43-63% lower in 2009 and 2011 while compared to Ahero in 1989/1990, biting rates in Asembo were >98% lower in 2009 and 2011. Similarly, outdoor biting rates (Table 3) were generally lower in 2009 and 2011 compared to previous reports. For *An. gambiae* s.l. and *An. gambiae* s.s., outdoor biting rates were estimated at 0.187 and 0.135 bites per person per night in Asembo in 2011. These figures were >90% lower compared to *An. gambiae* s.l. collected in Miwani in 1989/1990. Biting rates of *An. arabiensis* were 6.710 and 0.081 bites per person per night in Asembo in 2009 and 2011, respectively. These figures were 74% and 99% lower compared to those estimated for Ahero in 1989/1990. For *An. funestus*, outdoor biting rates were 0.86 in 2009 and 0.612 in 2011. These were higher compared to Asembo in 1985–1988 and Miwani in 1989/1990 but were >90% lower compared to Ahero in 1989/1990.

**Table 2:** **Indoor biting rates, sporozoite rates and EIRs by species, location and year**

	*An. gambiae* s.l.*			*An. gambiae*	*An. arabiensis*			*An. funestus*				
	Asembo, 1985-88 ^†^	Miwani, 1989-90 ^‡^	Asembo, 2011	Asembo, 2011	Miwani, 1989-90 ^‡^	Asembo, 2009	Asembo, 2011	Asembo, 1985-88 ^†^	Miwani, 1989-90 ^‡^	Ahero, 1989-90 ^‡^	Asembo, 2009	Asembo, 2011
Biting rate	3.3	7.625	0.225	0.211	47.58	7.785	0.141	0.6	3.417	98.04	1.935	1.262
Sporozoite rate	0.131	0.060	0.100	0.097	0.011	0.005	0.042	0.049	0.043	0.043	0.005	0.082
EIR (Daily)	0.432	0.458	0.023	0.020	0.523	0.039	0.006	0.029	0.147	4.216	0.010	0.103

*For Asembo 2011, *An. gambiae* s.l. represents those mosquitoes that were morphologically identified as *An. gambiae* s.l. but could not be identified by PCR.

^†^Estimated from man biting rate observed at Saradidi as reported in Table 1 of Beier et al. [27].

^‡^Daily biting rates were estimated by converting the total number of bites as reported by Githeko et al. [34] to nightly biting rates. Sporozoite rates presented are those reported by Githeko et al. [35] from the same villages and time period.

**Table 3:** **Outdoor biting, sporozoite rates and EIRs by species, location and year**

	*An. gambiae* s.l.*			*An. gambiae*	*An. arabiensis*			*An. funestus*				
	Asembo, 1985-88 ^†^	Miwani, 1989-90 ^‡^	Asembo, 2011	Asembo, 2011	Ahero, 1989-90 ^‡^	Asembo, 2009	Asembo, 2011	Asembo, 1985-88 ^†^	Miwani, 1989-90 ^‡^	Ahero, 1989-90 ^‡^	Asembo, 2009	Asembo, 2011
Biting rate	2.7	3.667	0.187	0.135	25.417	6.710	0.081	0.5	0.417	14.875	0.860	0.612
Sporozoite rate	0.132	0.060	0.065	0.088	0.011	0.005	0.018	0.070	0.043	0.043	0.005	0.090
EIR (Daily)	0.356	0.220	0.012	0.012	0.280	0.034	0.001	0.035	0.018	0.640	0.004	0.055

*For Asembo 2011, *An. gambiae* s.l. represents those mosquitoes that were morphologically identified as *An. gambiae* s.l. but could not be identified by PCR.

^†^Estimated from man biting rate observed at Saradidi as reported in Table 1 of Beier et al. [27].

^‡^Daily biting rates were estimated by converting the total number of bites as reported by Githeko et al. [34] to nightly biting rates. Sporozoite rates presented are those reported by Githeko et al. [35] from the same villages and time period.

In 2011, the overall sporozoite rate was 0.082 (n = 1,921). For *An. arabiensis*, sporozoite rates were 0.042 indoors (n = 95) and 0.018 outdoors (n = 55). For *An. gambiae* s.l., sporozoite rates were 0.100 indoors (n = 150) and 0.065 outdoors (n = 123) while for *An. gambiae* s.s., the sporozoite rates were 0.097 indoors (n = 144) and 0.088 outdoors (n = 91). Sporozoite rates were 0.082 among *An. funestus* collected indoors (n = 850) and 0.090 among those collected outdoors (n = 413). The sporozoite rates observed in 2009 were so low that an overall estimate of 0.005 (n = 224) was used for both *An. arabiensis* and *An. funestus* both indoors and outdoors. Sporozoite rates from 1985 to 1988 and 1989/1990 ranged from 0.060 to 0.132 for *An. gambiae* s.l. and from 0.043 to 0.070 for *An. funestus*. Sporozoite rates in *An. arabiensis* were 0.011 both indoors and outdoors in Ahero 1989/1990. Estimated daily EIRs generally followed the trends of biting rates although EIRs were lowest in 2009 due in part to the low sporozoite rates. Overall indoor EIRs were estimated at 0.462 infectious bites per person per night for Asembo in 1985 to 1988, 0.604 for Miwani in 1989/1990, 4.739 for Ahero in 1989/1990, 0.049 for Asembo in 2009 and 0.152 for Asembo in 2011.

### Indoor versus outdoor biting

**Figure 1:**
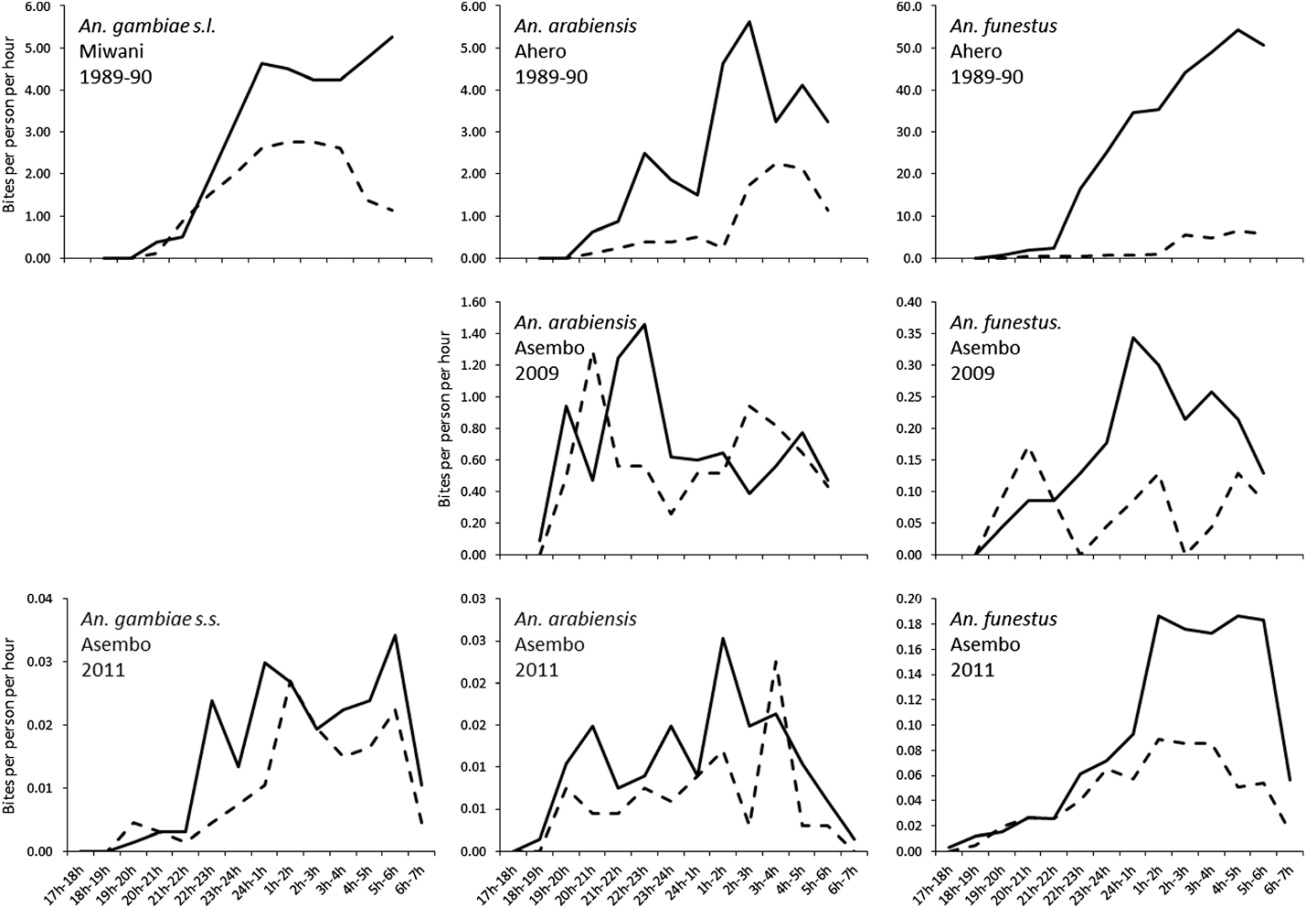
**Indoor (solid lines) and outdoor (dashed lines) biting rates by time of night for** ***An. gambiae*** **s.l.,** ***An. gambiae*** **s.s.,** ***An. arabiensis*** **and** ***An. funestus*** **at different studies sites and different years.**

The number of bites by hour and location are shown in Figure 1 while the ratio of indoor to outdoor biting is given in Table 4. The overall proportion of biting indoors versus outdoors by species and by year is listed in Table 5. For *An. gambiae* s.l., 65.5% of mosquitoes were captured biting indoors in Miwani in 1989/1990 compared to 61.3% in Asembo in 2011 (χ^2^ = 1.1, p = 0.288). For *An. arabiensis*, 75.6% were captured biting indoors in Ahero in 1989/1990 while in Asembo, 54.1% and 63.3% were captured indoors in 2009 and 2011, respectively (χ^2^ = 32.5, p < 0.001). For *An. funestus*, the proportion captured biting indoors fell from 92.3% in Ahero in 1989/1990 to 69.7% and 67.3% in Asembo in 2009 and 2011, respectively (χ^2^ = 414.3, p < 0.001).

**Table 4:** **Indoor/outdoor biting ratios, percent of mosquitoes biting before 2200, percent of the total mosquito bites that occurs while people are indoors (**π_**i**_ **) and percent of the total mosquitoes that occurs while people are sleeping (**π_**s**_ **)**

	*An. gambiae* s.l.		*An. gambiae*	*An. arabiensis*			*An. funestus*		
	Miwani, 1989-90	Asembo, 2011	Asembo, 2011	Ahero, 1989-90	Asembo, 2009	Asembo, 2011	Ahero, 1989-90	Asembo, 2009	Asembo, 2011
Indoor/Outdoor ratio	1.9	1.2	1.6	3.1	1.2	1.7	12.0	2.3	2.1
% biting before 2100 indoors	1.1	6.6	2.1	2.2	18.2	18.9	0.8	6.5	4.2
% biting before 2200 indoors	2.6	11.2	3.5	5.3	33.3	24.2	1.6	10.8	6.2
% biting before 2100 outdoors	0.7	11.1	5.5	1.4	25.3	14.5	1.4	30.2	8.2
% biting before 2200 outdoors	5.6	18.2	6.6	4.1	33.3	19.9	2.9	40.1	12.3
Non-users of nets:									
Exposure indoors (π_i_)	0.99	0.96	0.98	0.99	0.95	0.97	0.99	0.97	0.98
Exposure while asleep (π_s_)	0.98	0.90	0.95	0.97	0.80	0.84	0.98	0.91	0.94
Users of nets:									
Exposure indoors (π_i_)	0.96	0.72	0.83	0.97	0.80	0.87	0.99	0.78	0.86
Exposure while asleep (π_s_)	0.72	0.36	0.52	0.64	0.20	0.25	0.77	0.40	0.50

**Table 5:** **Proportion of indoor versus outdoor biting by species and location**

	Indoor	Outdoor	Total mosquitoes captured	χ^2^	P
*An. gambiae* s.l.				1.1	0.288
Miwani, 1989-90	65.5(60.8-70.1)	34.5(29.9-39.2)	411		
Asembo, 2011*	61.3(55.0-67.5)	38.7(32.5-45.0)	235		
*An. arabiensis*				32.5	<0.001
Ahero, 1989-90	75.6(70.7-80.5)	24.4(19.5-29.3)	299		
Asembo, 2009	54.1(48.9-59.3)	45.9(40.7-51.1)	355		
Asembo, 2011	63.3(55.6-71.0)	36.7(28.9-44.4)	150		
*An. funestus*				414.3	<0.001
Ahero, 1989-90	92.3(91.3-93.3)	7.7(6.7-8.7)	2725		
Asembo, 2009	69.7(58.6-80.8)	30.3(19.2-41.4)	66		
Asembo, 2011	67.3(64.7-69.9)	32.7(30.1-35.3)	1267		

Binomial confidence intervals are provided in parentheses.

*An. gambiae s.s.

### *Parity rates and sporozoite rates*vs. *time and location of biting*

Parity rates were estimated in 2011 by species and by time of night. Overall parity rates ranged from a low of 0.74 for *An. gambiae* s.l. collected indoors to a high of 0.91 for *An. arabiensis* outdoors. Parity rates were generally similar throughout the night and by χ^2^ test, there were no significant differences in parity rates by time of biting (p > 0.05 for all comparisons). However, overall numbers examined were low at each time point and χ^2^ statistics could not be estimated for *An. gambiae* s.l. or *An. gambiae* s.s. collected indoors. For *An. arabiensis*, sporozoite rates ranged from 0.005 in 2009 to 0.042 indoors in 2011 while sporozoite rates for *An. gambiae* s.l. ranged from 0.060 in Miwani in 1989–1990 (indoors and outdoors) to 0.131 in Asembo (indoors) in 1985–1988. Sporozoite rates for *An. gambiae* s.s. were only estimated in 2011 when they were 0.088 outdoors and 0.097 indoors. Sporozoite rates among *An. funestus* ranged from 0.005 in 2009 to 0.090 outdoors in 2011. Comparisons of sporozoite rates by time of night in 2011 found no statistically significant differences for any species (p > 0.05 for all comparisons).

### Time of biting

The number of bites per hour by year, species and location is presented in Figure 1. The proportion biting indoors at different periods of the night is given in Table 6. There was an increased proportion of biting that occurred in the period from 18 h to 21 h for *An. arabiensis* and *An. arabiensis*. For *An. gambiae* s.l., the proportion of indoor biting during this period rose from 1.1% of the total bites in Miwani in 1989/1990 to 2.2% in Asembo in 2011 (χ^2^ = 2.0, p = 0.565). Early indoor biting by *An. arabiensis* in Ahero in 1989/1990 accounted for 2.2% of the total bites compared to 18.2% and 19.1% in 2009 and 2011 (χ^2^ = 74.1, p < 0.001). For *An. funestus*, 0.8% of bites occurred in the early evening in Ahero in 1989/1990 while 6.5% and 4.4% of all indoor bites occurred during this time period in 2009 and 2011, respectively (χ^2^ = 59.9, p = 0 < 0.001).

**Table 6:** **Proportion of indoor biting that occurred at different times of night by species and location**

	1800 to 2100	2100 to 2400	0000 to 0300	0300 to 0600	Total mosquitoes captured	χ ^2^	P
*An. gambiae* s.l.						2.0	0.565
Miwani, 1989-90	1.1(0.0-2.4)	16.7(12.3-21.2)	39.8(33.9-45.6)	42.4(36.4-48.3)	269		
Asembo, 2011*	2.2(0.0-4.6)	21.2(14.3-28.0)	37.2(29.1-45.3)	39.4(31.2-47.6)	137		
*An. arabiensis*						74.1	<0.001
Ahero, 1989-90	2.2(0.3-4.1)	18.6(13.5-23.7)	41.6(35.1-48.0)	37.6(31.3-43.9)	226		
Asembo, 2009	18.2(12.7-23.7)	40.1(33.1-47.1)	19.8(14.1-25.4)	21.9(16.0-27.7)	192		
Asembo, 2011	19.1(11.2-27.1)	22.3(13.9-30.8)	35.1(25.4-44.8)	23.4(14.8-32.0)	94		
*An. funestus*						59.9	<0.001
Ahero, 1989-90	0.8(0.4-1.1)	14.0(12.7-15.4)	36.2(34.3-38.1)	48.9(47.0-50.9)	2515		
Asembo, 2009	6.5(0.0-13.7)	19.6(8.1-31.0)	43.5(29.1-57.8)	30.4(17.1-43.7)	46		
Asembo, 2011	4.4(3.0-5.8)	13.0(10.7-15.4)	37.6(34.3-41.0)	44.9(41.5-48.3)	813		

Binomial confidence intervals are provided in parentheses.

*An. gambiae s.s.

### Intersection between mosquito and human behavior

**Figure 2:**
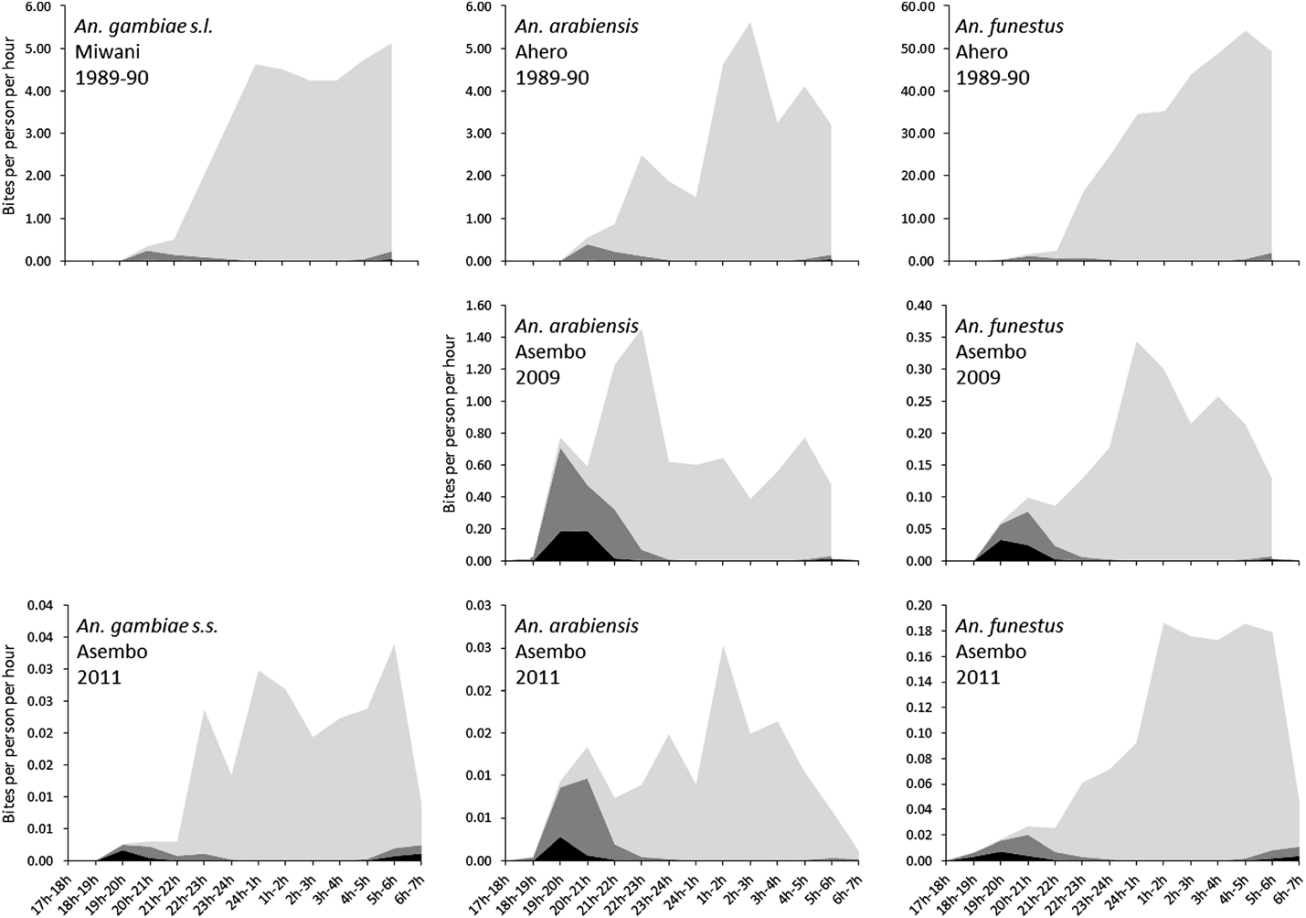
**Profiles of biting by** ***An. gambiae*** **s.l.,** ***An. gambiae*** **s.s.,** ***An. arabiensis*** **and** ***An. funestus*** **experienced by the human population at different studies sites and different years.** The black area represents biting that occurs outdoors, the dark grey represents biting that occurs indoors before people are asleep and the light grey represents biting that occurs while people are asleep.

Use of any net the previous night was 88.8% while use of an ITN the previous night was 74.3%. In surveys of human behavior, 85.4% of people reported going inside their house between 20 h and 21 h and 97.1% reported going inside their house between 21 h and 22 h while 24.8% of people reported going to bed between 20 h and 21 h and 72.6% reported going to bed between 21 h and 22 h. Using models described by Seyoum et al. [36], we estimated the exposure of persons according to their behavior and the time and location of mosquito biting. A graphical representation of the times and locations of human exposure is presented in Figure 2. The proportion of mosquito bites that occurred while people were indoors (π_i_) was 0.99 for all three species in 1989/1990 in Miwani and Ahero. In 2009 and 2011, these values declined only slightly, ranging from 0.95 to 0.98. Similarly, in 1989/1990, the proportion of bites occurring during the period that people were sleeping (π_s_) was high: 0.97 for *An. arabiensis* and 0.98 for *An. gambiae* s.l. and *An. funestus*. The proportion biting while people were asleep in 2009 and 2011 declined but was still high. For *An. gambiae* s.l. and *An. gambiae* s.s., π_s_ values were 0.90 and 0.95 in Asembo in 2011. Similarly, π_s_ values were 0.91 and 0.94 for *An. funestus* in Asembo in 2009 and 2011, respectively. The largest drops in π_s_ values occurred for *An. arabiensis.* Values of π_s_ were 0.80 in 2009 and 0.84 in 2011 indicating 80% of bites occurred while people were in bed in 2009 and 84% of bites occurred while people were in bed in 2011.

Assuming a personal protective efficacy of ITNs of 93.7%, π_i_ values were lower for ITN users but were still >0.80 for all species and years except for *An. funestus* in Asembo in 2009 when π_i_ was 0.78 and for *An. gambiae* s.l. in 2011 when it was 0.71. Values for π_s_ for ITN users were much lower for all species, particularly after ITNs were implemented. Values for π_s_ were >0.60 for all three species in 1989/1990 but were less than 0.50 for all species in 2009 and 2011 (Table 4).

## Discussion

The Asembo region of western Kenya has had a long history of ITN use, beginning with a large-scale ITN trial that began in 1997 [20, 21]. Since the end of the trial, high ITN coverage was maintained by the Kenya Medical Research Institute and the US Centers for Disease Control and Prevention through a free retreatment program and periodic net replacement. Beginning in 2004, the Kenya Division of Malaria Control began to scale up ITNs. Initial efforts focused on the distribution of heavily subsidized nets through government health clinics to children and pregnant women, the population considered most at risk. This was supplemented by a national mass campaign targeting children under 5 years of age in 2006 [38] and another mass campaign targeting universal coverage in 2011 [39]. At the time of the HLC study in 2011, nearly 90% of residents used a bed net the previous night and over 75% used an ITN. Estimated entomological inoculation rates were much lower than those estimated before the scale-up of ITNs. For several years after the national scale up of ITNs, *An. funestus* and *An. gambiae* s.s. were rare, while *An. arabiensis* was the predominant mosquito, presumably due to its ability to survive by feeding on cattle or other hosts [29], thereby avoiding contact with the insecticides in ITN fibers. Given the tremendous selective pressure upon the more anthropophilic *An. gambiae* and *An. funestus*, there was a statistically significant shift in the location and time of biting compared to the pre-ITN era, with a higher proportion of feeding occurring early in the evening in 2009 and 2011 compared to 1989/1990. However, these shifts were based on proportionate numbers. It is not clear if they represent real shifts in mosquito biting behavior or if eliminating a large amount of biting indoors late at night has made a pre-existing set of outdoor, early evening biting become more apparent. Furthermore, based on inferences that can be drawn from the π_i_ and π_s_ values, the majority of biting on non-users of nets still occurred indoors, late at night during the period that people were sleeping.

For net users, estimates of π_i_ and π_s_ were lower compared to non-users but most biting still occurred indoors and/or while people were asleep. ITNs were assumed to prevent over 90% of biting as estimated for east African *An. gambiae* and *An. funestus* by Okumu et al. [37] in well controlled experimental hut trials. However, these estimates likely represent a best case scenario. Net use may be inconsistent among many users so that those reporting use the previous night may not use them every night. Many surveys make the important distinction between measures of household ownership and actual use, although some of this disparity may be related to the lack of adequate bednets within the household for all sleepers each night. Even if residents use the nets every night, many may get up in the middle of the night, exposing themselves to mosquitoes. Furthermore, there are increasing reports from multiple sites that nets that are routinely used by residents of sub-Saharan Africa are in poor condition in terms of their physical integrity and their insecticidal activity [40–42]. Combined with increasing resistance to pyrethroids among both *An. gambiae* s.s. and *An. funestus*[2, 3], ITNs may provide less than optimal protection against mosquitoes and malaria transmission [43]. Thus, the π_i_ and π_s_ estimates for ITN users represent a theoretical minimum that could be achieved with current long-lasting insecticidal net technology. Even at these theoretical minimums, more than three quarters of biting occurred indoors for all species in Asembo and approximately half of the biting by *An. gambiae* s.s. and *An. funestus* occurred while people were asleep under nets. *Anopheles arabiensis*, a species which exhibits much more plasticity in its behaviors, was the only species which was much less likely to bite while people were asleep under nets although >80% of biting by this species on net users still occurred indoors.

Parity rates and sporozoite rates during the HLC collections were higher than observed in Asembo in the recent past. Bayoh et al. [29] reported that parity rates in Asembo in 2005 were significantly lower than Seme, a neighboring area with lower ITN coverage. By 2011, parity rates in Asembo had risen to the levels observed in Seme in 2005. Similarly, sporozoite infection rates in Asembo since the end of the ITN trial were consistently low in all the main vectors until the present study. The high rates of parous and sporozoite positive mosquitoes suggest a waning community protective effect of ITNs, although it is difficult to reconcile this conclusion with the observed, sustained low numbers of biting mosquitoes relative to the pre-ITN era. There was no evidence for an excess of nulliparous mosquitoes during the early evening hours, as was observed in Sierra Leone [44].

The issue of mosquito biting behavior and its relevance to vector control has long been a concern [45]. In Ethiopia before ITNs were introduced, the peak biting time of *An. arabiensis* was between 19 h and 20 h, suggesting ITNs might have limited impact in this area [46]. Subsequent studies conducted after the distribution of ITNs indicated no change in biting time although the impact of ITNs on malaria transmission was not reported [47]. During the era of large-scale trials of ITNs, studies to assess their impact on mosquito biting times had mixed results. In Tanzania [10] and coastal Kenya [9], significant changes in biting times were observed between villages with and without ITNs while other studies in the same countries produced equivocal results [11, 12]. Following the scale up of ITNs throughout sub-Saharan Africa, the assessment of vector biting behavior has not been included as part of routine entomological monitoring programs. Where studies have documented the biting times of vectors in the context of high ITN coverage, the results have again been mixed. In a comparison of 6 different sites from across Africa, Huho et al. [17] reported that only 40-63% of *An. gambiae* s.l. and 22-69% of *An. funestus* were captured biting indoors. However, when matched to human activity, π_i_ values ranged between 0.87 and 0.97 for *An. gambiae* s.l. and between 0.62 and 0.97 for *An. funestus*. It was concluded that while residual outdoor transmission after the scale up of ITNs and indoor residual spraying remains a concern, residual indoor transmission is likely the main driver of malaria transmission in sub-Saharan Africa even in areas with high coverage of ITNs [17, 48]. Exposure of humans to mosquito bites while sleeping was not reported by Huho et al. but a separate report from southern Zambia generated estimates of π_s_ for *An. quadriannulatus* and *An. funestus* that were similar to those observed in the current study. In contrast, recent reports of *An. funestus* biting behavior in west Africa are particularly alarming. In Benin, 26% of bites occurred after 6 h [15], while in Senegal a second peak of biting was observed between 8 h and 9 h with biting observed up until 11 h [16]. The rapid decline in biting rates of *An. funestus* in Asembo in 2011 from 5 h-6 h to 6 h-7 h suggests that this species follows expected behavioral patterns although no collections were carried out after 7 h and the possibility that additional biting occurs later in the morning cannot be completely ruled out.

The vector population in western Kenya has undergone dramatic shifts associated largely with the scale up of ITNs. Following the ITN trial in the late 1990s, *An. funestus* became rare [30] while *Anopheles gambiae*, despite increasing resistance to pyrethroids, became a minor vector in western Kenya beginning in 2007. With the predominance of *An. arabiensis*, it was hypothesized that behavioral plasticity of this vector allowed it to survive by avoiding mosquito nets but still maintain transmission [29]. It was therefore somewhat surprising that in 2011 the most common mosquito collected was *An. funestus* and that, of the *An. gambiae* s.l. identified by PCR, over 60% were *An. gambiae* s.s. The shift to more anthropophilic vectors may partially explain the rebound in malaria in Asembo. However, the rebound in malaria occurred in 2009, when HLC collections were predominantly *An. arabiensis,* which made up nearly 95% of PCR identified *An. gambiae* s.l. The rise of *An. funestus* and its presumed association with pyrethroid resistance has been reported elsewhere [30]. Pyrethroid resistance in *An. gambiae* has been reported from several sites in western Kenya though it is not clear what impact it has had on the effectiveness of ITNs. In Bungoma County, near the Kenya-Uganda border, pyrethroid resistant mosquitoes were frequently observed resting inside ITNs which were subsequently shown to have adequate insecticide to kill susceptible Kisumu strain mosquitoes [43]. However, in Gem—a site just north of Asembo—no anophelines were observed inside nets.

There are several limitations to conducting retrospective comparisons such as those presented here and the results should be interpreted with caution. First, the results compared across years were from studies that differed in several important ways that may have influenced the results. Collections carried out in 1989/1990 were done in areas that were approximately 60 km from Asembo. Even the study sites in Asembo differed from 2009 to 2011. Nightly biting rates and EIRs should particularly be viewed with caution as these are known to exhibit wide ranges of seasonal and inter-annual variability. Comparisons with Miwani in 1989/1990 should also be regarded with caution as the species composition of *An. gambiae* s.l. was not directly estimated in that year but were reported as 55% *An. gambiae* s.s. and 45% *An. arabiensis*[34]. While this introduces some uncertainty into the comparisons, the distribution of the biting curves by *An. gambiae* s.s. and *An. arabiensis* over the years were generally similar, although *An. arabiensis* was more likely to bite earlier after the introduction of ITNs. While HLCs were used for all collections, there were some methodological differences that may have influenced the results. For example, the collections from 1989/1990 were from twice monthly HLCs in each village over the course of an entire year. Collections in 2009 and 2011 were done over 24 to 30 nights during peak transmission. In 2011, outdoor collectors were allowed to stop in the event of rainfall, which occurred in 6.5% of man-hours of collection. This was not adjusted for in the analysis but would have resulted in an underestimate of the outdoor biting. However, estimates of π_i_ and π_s_ after removing outdoor person-hours, which were interrupted by rainfall did not change by more than 1%. While numerous studies have shown the patterns of mosquito biting to be similar to those observed in the present study [6, 7, 17], it cannot be ruled out that differences in location or season may have affected the diurnal biting pattern of the mosquitoes. Another issue with the HLC is that collectors are stationed at fixed points and are not reflective of the actual human population. In theory, this was adjusted for by estimating the proportion of biting when humans are indoors (π_i_) or indoors and sleeping (π_s_). However, mosquitoes that feed outdoors may have been seeking an indoor host but were diverted to a more convenient one that was stationed outdoors. Similarly, mosquitoes that were captured attempting to feed on one of the collectors during the night may have been unsuccessful in attempting to feed on a person protected by an ITN resulting in that mosquito feeding later in the night or, possibly, early the following night. Although ITN use was very high during the 2011 collections, their use in the households or rooms where HLCs were done was not documented at the time of the HLCs. The presence of a treated net may have affected mosquito behavior due to the excite-repellent effect of pyrethroids. For measures of human behavior, only one estimate was obtained from 2011 and assumed to be the same for all years. This may have slightly decreased the π_i_ and π_s_ values from 1989/1990 when it was reported that 90% of residents were indoors and in their beds by 21 h [34]. In 2011, approximately 85% of people were indoors by 21 h but only 25% were in bed. Lastly, the results of the statistical analysis and their biological implications should be considered carefully. In comparing biting times and locations, the original data from 1989/1990 were not available and analyses were therefore done with aggregate data and did not account for clustering, which resulted in much narrower confidence intervals. Had clustering been taken into account, it is possible that the differences would not have been statistically significant.

## Conclusions

There are increasing calls for new tools to address residual outdoor, early evening biting by mosquitoes that evade ITNs or IRS [4, 18]. However, data from western Kenya suggest that while some mosquitoes do bite outdoors, early in the evening, the majority of malaria transmission occurs indoors late at night, despite high usage of ITNs. Although this is not true for all malaria endemic settings and even in western Kenya, outdoor transmission will likely need to be addressed, these findings suggest that the development and implementation of other interventions targeting the indoor populations such as IRS [49, 50], insecticide treated wall liners [51], indoor spatial repellents [52], house screening [53] or other modifications to block mosquito entry into houses [54] could substantially reduce transmission in many areas of sub-Saharan Africa, including those where ITNs have been scaled up. Ideally, any strategy introduced to complement ITNs should be incorporated as part of an insecticide resistance management strategy [55] to address the growing threat of physiological resistance to pyrethroid insecticides, which at this time, appears to be a more urgent issue than that of behavioral resistance.
